# Ferulic Acid Exerts Anti-Angiogenic and Anti-Tumor Activity by Targeting Fibroblast Growth Factor Receptor 1-Mediated Angiogenesis

**DOI:** 10.3390/ijms161024011

**Published:** 2015-10-12

**Authors:** Guang-Wei Yang, Jin-Song Jiang, Wei-Qin Lu

**Affiliations:** Department of Vascular Surgery, Zhejiang Provincial People’s Hospital, Hangzhou 310003, China; E-Mails: guang2015nor@sina.com (G.-W.Y.); jinsong@163.com (J.-S.J.)

**Keywords:** ferulic acid, angiogenesis, melanoma, FGFR1, Akt

## Abstract

Most anti-angiogenic therapies currently being evaluated target the vascular endothelial growth factor (VEGF) pathway; however, the tumor vasculature can acquire resistance to VEGF-targeted therapy by shifting to other angiogenesis mechanisms. Therefore, other therapeutic agents that block non-VEGF angiogenic pathways need to be evaluated. Here, we identified ferulic acid as a novel fibroblast growth factor receptor 1 (FGFR1) inhibitor and a novel agent with potential anti-angiogenic and anti-cancer activities. Ferulic acid demonstrated inhibition of endothelial cell proliferation, migration and tube formation in response to basic fibroblast growth factor 1 (FGF1). In *ex vivo* and *in vivo* angiogenesis assays, ferulic acid suppressed FGF1-induced microvessel sprouting of rat aortic rings and angiogenesis. To understand the underlying molecular basis, we examined the effects of ferulic acid on different molecular components and found that ferulic acid suppressed FGF1-triggered activation of FGFR1 and phosphatidyl inositol 3-kinase (PI3K)-protein kinase B (Akt) signaling. Moreover, ferulic acid directly inhibited proliferation and blocked the PI3K-Akt pathway in melanoma cell. *In vivo*, using a melanoma xenograft model, ferulic acid showed growth-inhibitory activity associated with inhibition of angiogenesis. Taken together, our results indicate that ferulic acid targets the FGFR1-mediated PI3K-Akt signaling pathway, leading to the suppression of melanoma growth and angiogenesis.

## 1. Introduction

Melanoma is the most aggressive form of skin cancer and frequently chemo-resistant, caused by malignant transformation of melanocytes, which are pigment-producing cells mainly found in the skin and eyes [1]. Although early stages of melanoma can be successfully treated by surgical resection of the tumor, there is still no effective treatment for melanoma due to sustained and excessive angiogenesis [2]. Angiogenesis, considered crucial for the transition of tumors from a dormant to malignant state, is now established as one of the hallmarks of melanoma and responsible for over 90% of all cancer deaths [3]. Tumor growth is angiogenesis dependent, and therapy targeting tumor vasculature is an attractive alternative or adjunct to conventional therapy. Disrupting tumor angiogenesis has been shown effective for tumor growth and metastasis inhibition. Therefore, defining agents that inhibit melanoma angiogenesis may aid the development of more effective therapeutic strategies for combating melanoma [4].

Vascular endothelial growth factor (VEGF) was first described as a molecule that could increase the permeability of blood vessels [5]. Additionally, VEGF-A promotes the proliferation of new blood vessels and is essential for normal embryonic development. Besides VEGF-A, there is a family of proteins that include placenta growth factor (PIGF), VEGF-B, VEGF-C, VEGF-D and VEGF-E, which directly participate in the genesis of blood capillaries and lymphatic vessels [6]. Furthermore, recent studies have identified FGF1 as a direct activator of phosphatidyl inositol 3-kinase (PI3K)-protein kinase B (Akt), which is a key stimulus known to initiate endothelial cell migration, invasion and differentiation [7]. The mammalian target of rapamycin (mTOR), a serine/threonine kinase, is a downstream target of FGFR1, and it plays a critical role in cell survival and proliferation. Recent studies have suggested that the PI3K might play a vital role in tumor angiogenesis. Akt is a pivotal downstream target of PI3K during angiogenesis. Akt regulates multiple cellular processes, including tumor angiogenesis, cell cycle progression, cell growth, cell migration and cell metabolism [8]. Therefore, the PI3K/Akt signaling cascade plays a vital role in tumor angiogenesis. Inhibiting activated PI3K/Akt signaling contributes to angiogenesis inhibition, tumor growth arrest and metastasis suppression [9].

Currently, several strategies have been already reported to block the action of the kinase signaling pathway, including natural compounds and small molecules [10]. Phytochemicals are potential novel leads for developing anti-angiogenic drugs. Ferulic acid (4-hydroxy-3-methoxycinnamic acid) (FA), an effective component of many Chinese medicinal herbs, like Cimicifuga heracleifolia, Angelica sinensis and Lignsticum chuangxiong, is a ubiquitous phenolic acid in the plant kingdom [11]. FA exhibits many physiological functions including anti-oxidant, anti-microbial, anti-thrombosis, anti-inflammatory, anti-hypercholesterolemic, and anti-cancer activities [12]. FA molecules can inhibit cancer development by either scavenging reactive oxygen species, or by being involved in the cell cycle upon cellular uptake [13], or they show anti-proliferative and anti-metastasis effects by specifically inhibiting anti-apoptotic proteins, like Bcl-XL and Bcl2 [14]. FA has been reported to be able to function to induce differentiation and apoptosis in leukemia and lung cancer and to suppress tumor angiogenesis and cell metastasis. In the past, few studies have reported that FA serves as an angiogenic agent to augment angiogenesis, both *in vitro* and *in vivo*. This effect might be observed through the modulation of VEGF, platelet-derived growth factor (PDGF) and hypoxia-inducible factor-1 alpha (HIF-1α) [15]. Furthermore, ferulic acid promotes endothelial cell proliferation through upregulating cyclin D1 and VEGF [16]. However, other studies find that FA inhibits endothelial cell proliferation through nitric oxide (NO), downregulating the extracellular-regulated protein kinases1/2 (ERK1/2) pathway [17]. So far, the targets for anti-angiogenesis of FA and the effect of FA on melanoma growth and tumor angiogenesis are yet to be fully elucidated. In this study, the effects of FA on inhibiting angiogenesis and melanoma cell B16F10 proliferation were validated *in vitro* and *in vivo*. A mechanistic study further indicated that FA could significantly inhibit FGF1-induced FGFR1 phosphorylation and activation of downstream signaling transduction mediators, both *in vitro* and *in vivo*. Taken together, our data suggested that FA could function as a novel potent FGFR1 inhibitor that suppresses tumor angiogenesis and melanoma growth.

## 2. Results and Discussion

### 2.1. Kinase Inhibition Profile of Ferulic Acid (FA)

In this study, we screened the effect of ferulic acid (FA) on different kinases and found that FA exerted an FGFR1 activity inhibition property at a low dose. The kinase inhibitory activity was measured by using of radiometric assays, which were provided by Kinase Profile Service (Millipore, Darmstadt, UK). As shown in Table 1, FA exhibited great inhibitory activity on FGFR1 with an inhibitory rate of 92% at 1 µM. In addition, the inhibitory activity of FA was examined against FGFR2, because of its structural similarity to FGFR1. However, FA showed a relatively low inhibitory rate of 64%, 4%, 1% and 2% against FGFR2, VEGFR2, platelet-derived growth factor receptor-α (PDGFR-α) and PDGFR-β at 1 µM, respectively. Moreover, excellent selectivity for FGFR2 was evident compared to a range of unrelated tyrosine and serine/threonine kinases, including fms-like tyrosine kinase 3 (Flt3), c-Met, c-Kit, epidermal growth factor receptor (EGFR), c-RAF, *etc*.

**Table 1:** *In vitro* profile of ferulic acid (FA) against a panel of 20 kinases. The assays were performed in three independent experiments. Data are the means ± SD.

Kinase	Inhibition Rate at 1 µM (%)
FGFR1	92 ± 3
FGFR2	64 ± 1
VEGFR2	4 ± 0
Flt3	8 ± 2
PDGFR-α	1 ± 0
PDGFR-β	2 ± 0
c-Kit	6 ± 1
Aurora-A	−2 ± 2
Haspin	7 ± 2
ErbB4	10 ± 2
IKKβ	−5 ± 2
c-Met	11 ± 0
CDK2	−13 ± 2
PI3K	20 ± 1
EGFR	4 ± 1
JNK	−9 ± 0
mTOR	5 ± 2
GSK3β	4 ± 0
c-RAF	18 ± 4
JAK	5 ± 1

### 2.2. FA Inhibits Fibroblast Growth Factor 1 (FGF1) and Induces Human Umbilical Vein Endothelial Cells (HUVEC) Growth

**Figure 1:**
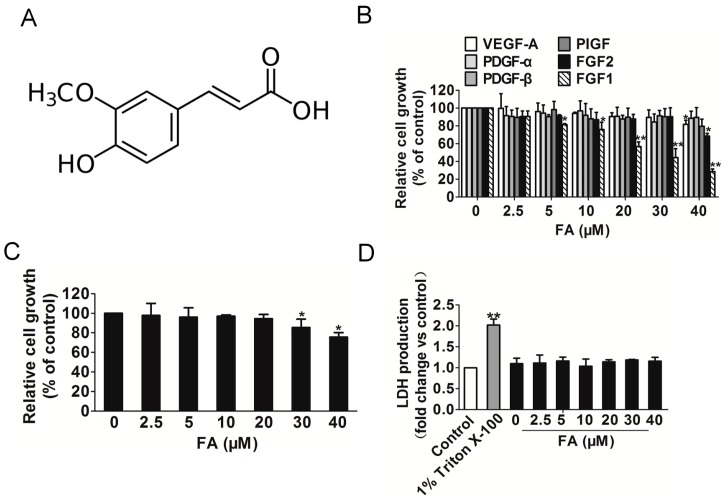
The effect of FA on HUVEC growth induced by FGF1. (**A**) The chemical structure of ferulic acid (FA); (**B**) the proliferation of HUVEC stimulated by FGF1 was significantly decreased by FA in a dose-dependent manner, while FA had little inhibitory effect on HUVEC that were stimulated by other angiogenesis stimulates. Data are from three independent experiments and are the mean ± SD. *n =* 3, * *p* < 0.05, ** *p* < 0.01 *vs.* the control; (**C**) FA had little inhibitory effect on HUVEC in the absence of FGF1. Data are from three independent experiments and are the mean ± SD. *n =* 3, * *p* < 0.05 *vs.* 0 μM FA treatment; (**D**) FA administration did not result in LDH release, indicating that FA brought little toxic effect on HUVEC. Data are from three independent experiments and are the mean ± SD. *n =* 3, ** *p* < 0.01 *vs.* the control.

To systematically assess the anti-angiogenic effect of FA (Figure 1A) on endothelial cells, the cell viability of HUVEC was determined by the 3-(4,5)-dimethylthiahiazo(-z-y1)-3,5-di-phenytetrazoliumromide (MTT) assay. We initially sought to evaluate the inhibitory activity of FA on endothelial cell proliferation and to evaluate the specificity of this effect for distinct angiogenic stimuli, including VEGF-A, FGF1, FGF2, PDGF-α, PDGF-β or phosphatidylinositol-glycan biosynthesis class f protein (PIGF). As shown in Figure 1B, the proliferation of HUVEC stimulated by FGF1, rather than other angiogenic stimuli, was markedly decreased after FA treatment ranging from 5–40 μM for 24 h, indicating that extracellular FGF1 acted as a strong stimulus for HUVEC proliferation. To determine the concentration of FA that does not induce cytotoxicity in HUVEC in the absence of FGF1, HUVEC were initially treated with FA (2.5–40 μM) for 24 h, and the cell viability was evaluated by the MTT assay. As shown in Figure 1C, FA did not exert significant cell viability up to 20 μM, but over 30 μM FA exhibited a cytotoxic effect in HUVEC compared to the control. To validate whether FA would result in toxicity effects on HUVEC, the lactate dehydrogenase (LDH) cytotoxicity assay was carried out. As shown in Figure 1D, Triton X-100 significantly increased LDH release, and FA brought little toxic effects on HUVEC when compared to the vehicle control. Therefore, further analyses of the biological activities of FA were performed using less than a 10 μM concentration of FA in endothelial cells.

### 2.3. FA Inhibits FGFR1 Kinase Activity in HUVEC

**Figure 2:**
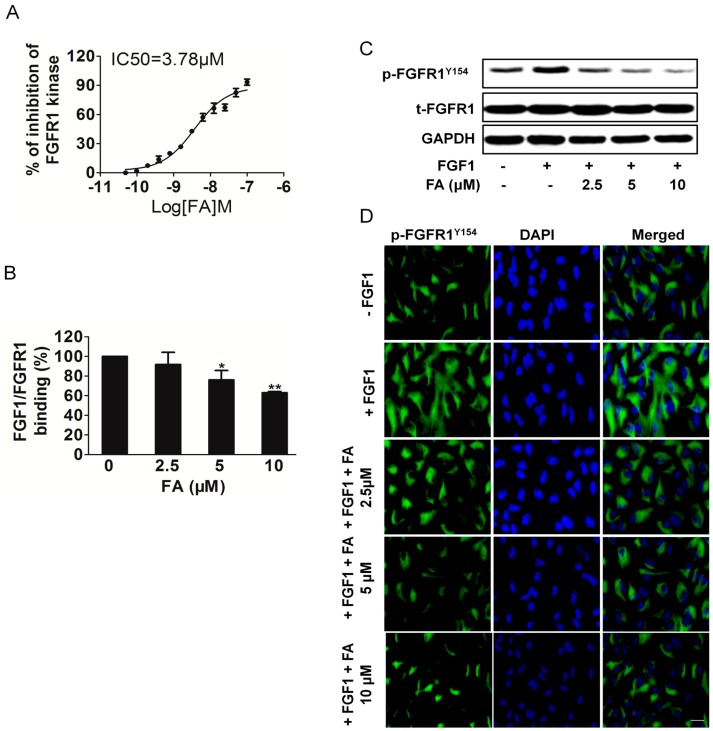
FA inhibits FGFR1 binding with FGF1 and attenuates FGFR1 tyrosine kinase activity. (**A**) Inhibition of FGFR1 kinase activity by FA was analyzed. Data are expressed as the percentages of the vehicle control. Data are from three independent experiments and are the mean ± SD. *n =* 3; (**B**) Effect of FA on the binding of FGFR1 to immobilized FGF1. Data are from three independent experiments and are the mean ± SD. *n =* 3, * *p* < 0.05, ** *p* < 0.01 compared to the control; (**C**) Western blot analysis of effect of FA on phosphorylation of FGR1. HUVEC were pre-treated with FA followed by stimulation with FGF1 for 2 min; p-FGFR1^Y154^ and GAPDH were assayed; (**D**) Immunofluorescent staining analysis of the effect of FA on FGFR1 expression in HUVEC. Cells were treated with FA under FGF1. The green color represents detection of p-FGFR1^Y154^, while nuclei were counterstained with blue color using DAPI (the scale bar represents 50 μm).

To confirm whether FA decreased the kinase activity of FGFR1, we performed an *in vitro* kinase assay with different concentrations [18]. Our data demonstrated that FA directly inhibited FGFR1 kinase activity in a dose-dependent manner with an IC_50_ of ~3.78 μM (Figure 2A). Moreover, FA inhibited FGFR2 kinase activity with an IC_50_ of ~12.5 μM, which demonstrated that FA was a selective FGFR1 inhibitor (Figure S1A). In addition, we compared the IC_50_ between FGFR1 inhibitor SSR128129E and FA. As shown in Figure S1B, FA exhibited effective activity to inhibit FGFR1 kinase as SSR128129E [18].

Further, we investigated whether FA inhibited the binding of FGF1 to its receptors FGFR1 and FGFR2. As shown in Figure 2B, FA decreased the binding of FGFR1 to immobilized FGF1. However, FA did affect the binding between FGF1 and FGFR2, but it did not reach a significant level (Figure S2A). Immunoprecipitation-Western blot analysis using HUVEC revealed that FA appeared to decrease FGF1 binding to FGFR1, rather than binding FGFR2 (Figure S2B). Previous studies suggested that blockage of FGFR1 activity could significantly limit the tumoral neovascularization process [18]. We then examined the effects of FA on phosphorylation of FGFR1 in HUVEC. We found that FGFR1 was strongly phosphorylated by exogenous FGF1 to HUVEC, while treatment with FA significantly blocked FGF1-induced phosphorylation of FGFR1 in a dose-dependent manner without affecting overall FGFR1 expression levels (Figure 2C). The effects of FA on FGF1 induced expression of FGFR1 phosphorylation in HUVEC were also examined using immunofluorescence analysis. Consistently, HUVEC treatment with FA (5 μM) in the presence of FGF1 hardly decreased the expression of p-FGFR1^Y154^ (Figure 2D). All of these results indicated that FA was a potent FGFR1 inhibitor. In order to verify further the dependency of FA inhibition on FGFR1, we performed siRNA-mediated knockdown experiments (Figure S3A). As expected, FGFR1 knockdown led to growth inhibition in the HUVEC (Figure S3B). However, FA-suppressed proliferation in HUVEC was abolished by FGFR1 siRNA, which suggests that FA inhibited HUVEC growth, dependent on FGFR1 (Figure S3C).

### 2.4. FA Inhibits FGF1-Induced Migration, Invasion and Tubular Structure Formation of HUVEC

**Figure 3:**
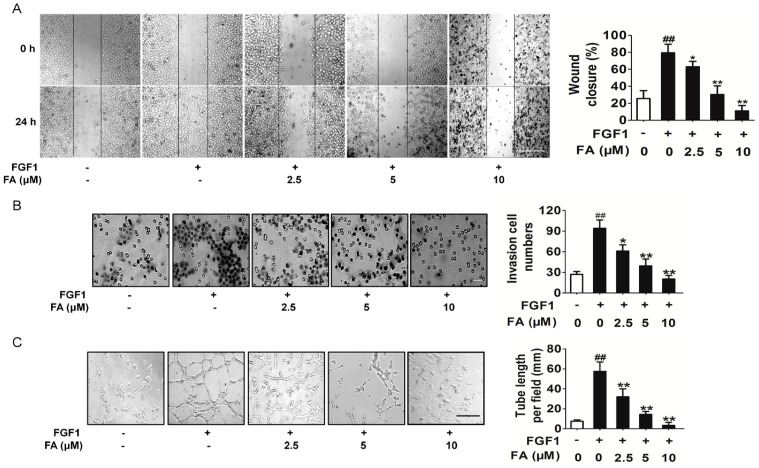
Effects of FA on HUVEC migration and invasion. (**A**) Effects of FA on HUVEC migration in wound migration assays (the scale bar represents 100 μm). Data are from three independent experiments and are the mean ± SD. *n =* 3, ^##^ *p* < 0.01 compared to the control, * *p* < 0.05, ** *p* < 0.01 compared to the FGF1 alone treatment; (**B**) FA decreased the number of invasive cells in a dose-dependent manner (the scale bar represents 50 μm). Data are from three independent experiments and are the mean ± SD. *n =* 3, ^##^ *p* < 0.01 compared to the control, * *p* < 0.05, ** *p* < 0.01 compared to the FGF1-alone treatment; (**C**) FA could dose-dependently suppress the capillary lengths of FGF1-stimulated HUVEC (the scale bar represents 100 μm). Data are from three independent experiments and are the mean ± SD. *n =* 3, ^##^ *p* < 0.01 compared to the control, ** *p* < 0.01 compared to the FGF1-alone treatment.

Cell migration is an essential step for endothelial cells to form blood vessels in angiogenesis [18]. The wound healing assay and transwell invasion assay were utilized to investigate the inhibitory effects of FA on the motility of HUVEC. The results showed that FA inhibited FGF1-induced HUVEC migration (Figure 3A) and invasion (Figure 3B) in a dose-dependent manner. The maturation of migrated endothelial cells into a capillary tube is a critical step during angiogenesis [19]. To examine the potential effects of FA on the tubular structure formation, we conducted two-dimensional matrigel assays and examined FA’s effect on tubular structure formation in HUVEC. FGF1 significantly enhanced the tubular network formation compared to HUVEC alone; however, treatment with FA strongly inhibited the FGF1-stimulated tubular network formation (Figure 3C). Overall, these results indicated that FA could suppress FGF1-induced angiogenesis by inhibiting migration, invasion and tube formation of HUVEC.

### 2.5. FA Inhibits Angiogenesis ex Vivo and in Vivo

**Figure 4:**
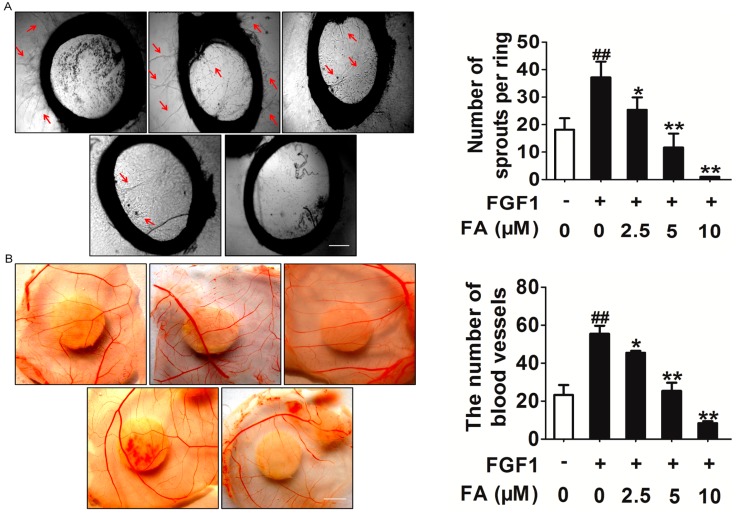
FA inhibits FGF1 induces angiogenesis *in vitro* and *in vivo*. (**A**) FA dose-dependently suppressed sprout formation on the organotypic model of rat aortic ring. Red arrows represent microvessel sprouting. The scale bar represents 1 mm. Data are from three independent experiments and are the mean ± SD. *n =* 3, ^##^ *p* < 0.01 compared to the control, * *p* < 0.05, ** *p* < 0.01 compared to the FGF1-alone treatment; (**B**) Chorioallantoic membrane (CAM) assay. Photo-pictographs of a typical experiment showing the angiogenesis pattern in different treatments. The scale bar represents 0.5 cm. Data are from three independent experiments and are the mean ± SD. *n =* 3, ^##^ *p* < 0.01 compared to the control, * *p* < 0.05, ** *p* < 0.01 compared to the FGF1-alone treatment.

Next, we confirmed the anti-angiogenic potential of FA *in vitro* and *in vivo*. Two well-established angiogenesis models, chicken chorioallantoic membrane (CAM) and the rat artic ring assay, were used *ex vivo* and *in vivo*. We determined the effects of FA on microvessel sprouting *ex vivo* using the rat aortic ring assay. Our results showed that FA almost completely inhibited FGF1-induced sprouting from the aortic rings (Figure 4A). Furthermore, in the chick embryo CAM assay, FGF1 could significantly induce neovascularization, whereas treatment with FA potently inhibited FGF1-induced neovascularization (Figure 4B). Dead embryos were not observed in the tested dose ranges of FA, indicating that FA-mediated anti-angiogenesis *in vivo* was not due to its toxicity.

### 2.6. FA Inhibits Activation of PI3K/Akt Signaling Induced by FGF1 in HUVEC

**Figure 5:**
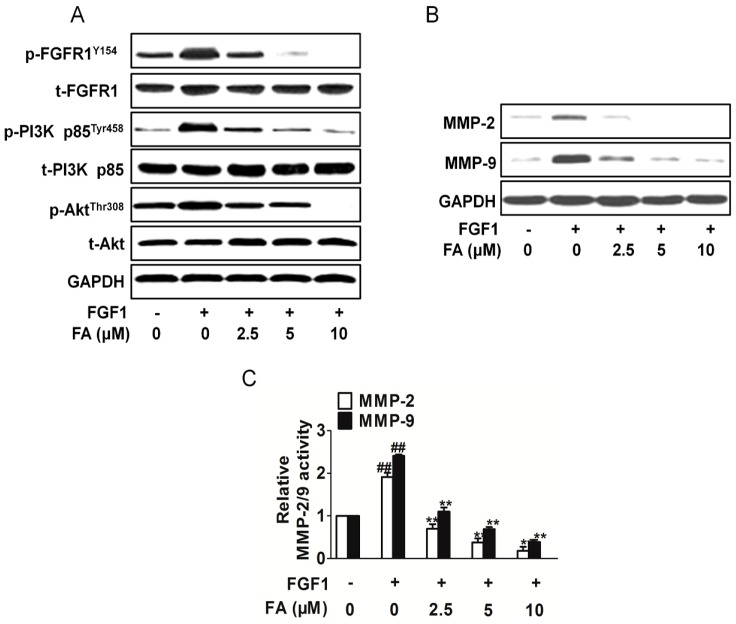
FA attenuates the FGFR1 signaling pathway. (**A**) FA inhibited the FGFR1 downstream signaling pathway PI3K/Akt in HUVEC. Blots are representative of three experiments. Each has the expression of GAPDH as the internal control; (**B**) HUVEC were exposed to FA in the presence of FGF1. Then, the MMP-2 and MMP-9 expression was analyzed by Western blots; (**C**) Quantification of MMP-2/9 activity in HUVEC treatment with FA in the presence of FGF1. Data are from three independent experiments and are the mean ± SD. *n =* 3, ^##^ *p* < 0.01 compared to the control, * *p* < 0.05, ** *p* < 0.01 compared to the FGF1-alone treatment.

To further understand the molecular basis of the FA-mediated anti-angiogenic activity, we next examined the modulation of FGF1-stimulated cellular signaling pathways in HUVEC. As shown in Figure 5A, the treatment of the HUVEC with FGF1 activated PI3K and Akt, but FA markedly suppressed the FGF1-induced phosphorylation of PI3K and Akt. The activation of ERK and mTOR was not greatly affected by FA (Figure S4A). Taking into account that matrix metalloproteinases (MMPs), such as MMP-2 and MMP-9, can be involved in the development of several human malignancies, as the degradation of collagen IV in the basement membrane and extracellular matrix facilitates tumor progression, including invasion, metastasis and angiogenesis, we analyzed their expression. Consistently, FA treatments significantly inhibits MMP-2 and MMP-9 expression stimulated by FGF1 (Figure 5B). Quantification of MMP-2 and MMP-9 activities using a fluorogenic assay showed a significantly decrease in extracellular MMP-2 and MMP-9 activity in FA-treated HUVEC (Figure 5C). These data suggest that the inhibition of the migration, proliferation and tube formation of the HUVEC is in part associated with the suppression of the FGF2-stimulated activation of the PI3K/Akt/MMPs signaling pathway. To validate that FA exerted its anti-angiogenesis effects through the FGFR1 signaling pathway exclusively, we assayed the FGFR2 signaling pathways in HUVEC treated with FA. As shown in Figure S4B, there were no apparent changes on FGFR2 activity in HUVEC treatment with FA.

### 2.7. FA Inhibits HUVEC Invasion Dependent on PI3K/Akt Signaling

**Figure 6:**
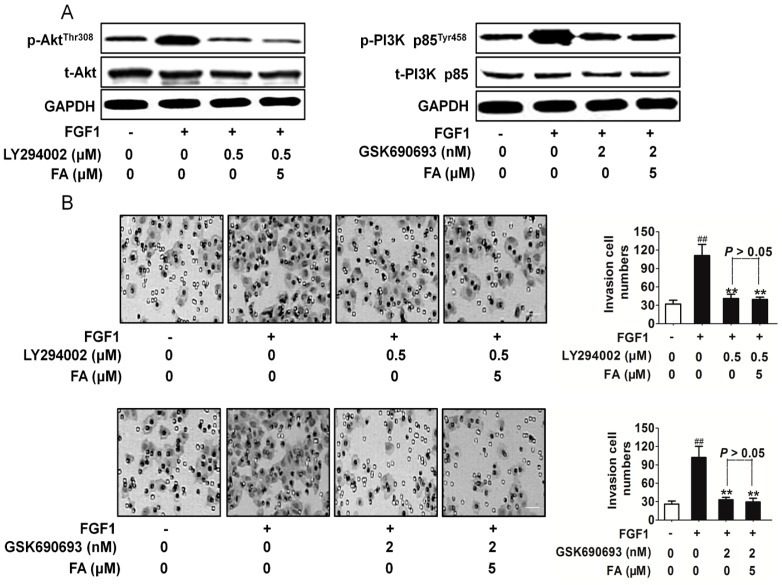
The effect of FA on HUVEC invasion dependent on FGFR1-mediated downstream signaling. (**A**) In the presence of GSK690693 or LY294002, protein extracts were analyzed by Western blot with antibody against phosphorylated Akt (Thr308) or p-PI3K p85^Tyr458^. The western blot assay was conducted to evaluate the PI3K and Akt activity inhibited by FA in the presence of the inhibitor; (**B**) in the presence of GSK690693 (2 nM) or LY294002, the invasion assay was conducted to evaluate the cell invasive ability. Data are from three independent experiments and are the mean ± SD. *n =* 3, ^##^ *p* < 0.01 compared to the control, ** *p* < 0.01 compared to the FGF1-alone treatment. The scale bar represents 50 μm.

To further confirm the association of FA with the Akt and PI3K signaling pathway, the effects of PI3K inhibitor LY294002 and Akt inhibitor GSK690693 were evaluated [20,21]. As shown in Figure 6A, FA exhibited a similar PI3K and Akt activity suppression pattern with the PI3K and/or Akt inhibitor in HUVEC. In addition, the suppression of the PI3K or Akt inhibitor on HUVEC invasion (Figure 6B) and angiogenesis (Figure S5) stimulated by FGF1 was not enhanced by FA. These data suggest that the suppression of the endothelial cell angiogenesis by FA is in part mediated by the downregulation of PI3K/Akt signaling.

### 2.8. FA Inhibits Melanoma Cell Proliferation and the FGFR1 Downstream Signaling Pathway

**Figure 7:**
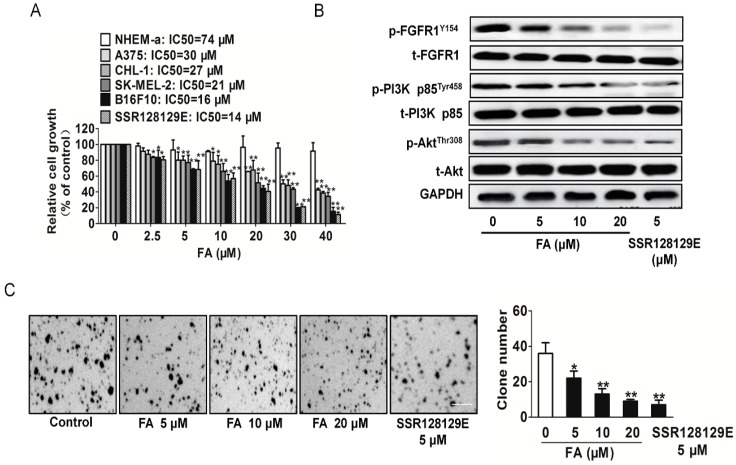
Inhibitory effects of FA on melanoma cells. (**A**) Melanoma cells were exposed to the indicated concentrations of FA for 24 h. Cell viability was determined by the MTT assay. The values are expressed as the percentage of viable cells normalized to the percentage of viable cells in 0.1% DMSO-treated cells. Data are from three independent experiments and are the mean ± SD. *n =* 6, * *p* < 0.05, ** *p* < 0.01 compared to the control; (**B**) FA inhibited FGFR1 downstream signaling molecules, including p-PI3K/PI3K and p-Akt/Akt in a dose-dependent manner. Blots are representative of three experiments. Each has the expression of GAPDH as the internal control; (**C**) FA inhibited anchorage-independent growth of B16F10 cells. B16F10 cells were grown for three weeks in 0.5% agarose gel containing vehicle or FA. The number of colonies lager than 2 mm in diameter was counted, and the data represent the means ± SD of three independent experiments, each performed in duplicate. * *p* < 0.05, ** *p* < 0.01 *vs.* the vehicle. Scale bars: 20 mm.

To access the anticancer activities of FA, four melanoma cell lines A375, CHL-1, SK-MEL-2 and B16F10, as well as normal melanocyte cells NHEM-a were used. As shown in Figure 7A, we found that FA and FGFR1 inhibitor SSR128129E inhibited melanoma cell proliferation in a dose-responsive manner. IC_50_ values from each cancer cell line and the incubation time were calculated. We also noted that the inhibitory effect on NHEM-a was maintained at higher micro-molar concentrations than the effect of equivalent doses of FA in melanoma cell. Collectively, these data demonstrate that FA has universal anti-cancer activity in melanoma cells and especially inhibited B16F10 cell growth.

To verify whether FA could inhibit anchorage-independent growth of B16F10 cells, we performed soft agar colony formation assays. FA greatly decreased, in a dose-dependent manner, the number and the size of colonies of B16F10 cells grown in soft agar as SSR128129E (Figure 7B), suggesting that FA inhibited the *in vitro* transformation capacity of B16F10 cells. As PI3K and Akt are reported to be downstream signals of FGFR1 and are also involved in tumor growth, we detected the PI3K and Akt by Western blot. The results showed that the PI3K and Akt activities were significantly reduced after FA administration (Figure 7C). To verify that FA exerted its anti-tumor growth effects through the FGFR1 signaling pathway exclusively, FGFR1 siRNA or FGFR2 siRNA plasmid was transfected into B16F10 cells. As expected, FA-suppressed proliferation in B16F10 cells was not neutralized by FGFR2 siRNA and was abolished by FGFR1 siRNA, which suggests that FA inhibited tumor growth dependent on FGFR1 (Figure S6).

### 2.9. FA Inhibits Tumor Growth and Angiogenesis in a B16F10 Cell Xenograft Model

**Figure 8:**
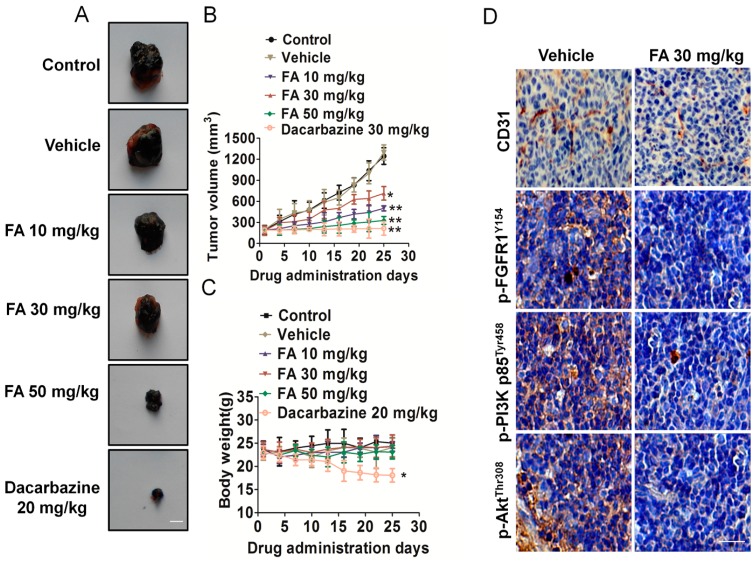
FA inhibits melanoma cell growth and tumor angiogenesis *in vivo*. (**A**) Representative mice with B16F10 xenografts and tumor masses. Scale bars: 0.5 cm; (**B**) Treatment with FA resulted in significant tumor growth inhibition *vs.* vehicle-treated mice. Values represent the means ± SD, *n* = 6, * *p* < 0.05, ** *p* < 0.01 *vs.* the control group; (**C**) Body weight changes in FA- and vehicle-treated mice. There was no significant difference in body weight between the FA- and vehicle-treated group, * *p* < 0.05 *vs.* the control group; (**D**) Tumor tissues were prepared for immunohistochemistry detection with antibodies against p-FGFR1^Y154^, p-PI3K p85^Tyr458^, p-Akt^Thr308^ and CD31. The scale bar represents 50 μm.

To evaluate the effects of the formation on melanoma growth and tumor angiogenesis *in vivo*, we further constructed a therapeutic experiment using a B16F10 cell xenograft mouse model. Representative mice with B16F10 xenografts and tumor masses are shown in Figure 8A. It was found that dacarbazine (positive control group) dramatically suppressed tumor volumes, and the dacarbazine-treated group was significantly inhibited compared to the vehicle group (Figure 8B). We found that intragastric administration of FA markedly inhibited tumor volume and tumor weight, as compared to the counterparts treated with DMSO. The average tumor volume of solid tumors in FA-treated mice was 714 ± 96 mm^3^ (10 mg/kg), 500 ± 36 mm^3^ (30 mg/kg) and 328 ± 56 mm^3^ (50 mg/kg) (Figure 8B). Furthermore, FA treatment was well tolerated, and there was no significant difference in weight between the vehicle group and the FA-treated groups (Figure 8C).

To further examine whether FA could suppress melanoma cell growth by inhibiting angiogenesis, tumor tissues were stained with specific antibodies against CD31 and p-FGFR1^Y154^. FA-treated mice showed a significant reduction of p-FGFR1^Y154^-positive cells in tumors. Tumor sections stained with anti-CD31 antibody revealed that FA inhibited tumor angiogenesis (Figure 8D). In addition, FA treatment also resulted in downregulation of FGFR1 downstream molecules’ phosphorylation, including Akt and PI3K (Figure 8D). Collectively, these data demonstrated that FA played an important role in suppressing angiogenesis, at least partly through FGFR1 signaling pathways.

### 2.10. Discussion

Human malignant melanoma is highly aggressive with a poor prognosis and high resistance to all standard anticancer therapies [1]. The importance of tumor angiogenesis in melanoma progression is underscored by the fact that it is an important target for the development of anticancer therapies based on the inhibition of angiogenesis, and anti-angiogenic therapy is now considered as the fourth strategy to treat cancer [4]. Cancer cells secrete numerous angiogenic factors, including VEGF-A, FGF1, EGF, PDGF, *etc.*, which play pivotal roles in the development of tumor angiogenesis by stimulating endothelial cell proliferation, migration and capillary tube formation [22]. Among all angiogenic factors, VEGFA is a well-known master switch of the angiogenic program. The role of VEGF and VEGFR-2 in tumor growth, maintenance and spread is well established, but other angiogenic factors switch on during cancer progression and induce resistance to VEGFR inhibitor monotherapy [23]. In fact, many previous studies report a positive role for the FGF1 pathway in directly controlling tumor angiogenesis. The binding of FGF1 to the FGF1 receptor initiates an intracellular signaling cascade PI3K/Akt and enhances the endothelial cell proliferation and migration, contributing toward cancer progression. Furthermore, activation of the intracellular PI3K/Akt signaling pathway by FGF1 results in the expression of various genes involved in cancer growth and development [24].

The identification of new drugs from natural products has a long and successful history. In the present work, we introduce a natural compound, ferulic acid, which is a phytochemical found in many fruits and vegetables, exhibits a broad range of therapeutic effects on human diseases, including diabetes and cancer, and has prominent anti-angiogenesis activity. In this study, we demonstrated for the first time that ferulic acid (FA) reduced FGFR1 activity at a low dose, which is close to selective FGFR1 inhibitor SSR128129E, and the inhibitory effect on FGFR2 kinase activity was maintained at a high half-maximum inhibitory concentration. Protein–protein interactions and regulation of the signal transduction circuitry play pivotal roles in tumor angiogenesis, as well as angiogenesis. The functions of vascular endothelial cells principal rely on FGFR1 signaling, and FGFR1 phosphorylation initiates downstream signaling pathways. In this study, we identified that FA disrupts the FGF1 interaction with its receptor FGFR1.

Angiogenesis is a complex process that occurs by a series of complex events, including endothelial cell migration and invasion [25]. Cell biology studies proved that FA is a small molecule inhibitor of FGF/FGFR1. As shown by a cell proliferation bioassay, wound healing and transwell invasion assays, FA effectively inhibited the growth, migration and invasion of HUVEC stimulated by FGF1. This inhibitory effect was also supported by the suppressed blood vessel formation and microvessel sprouting in chicken chorioallantoic membrane (CAM) and the rat artic ring assay. Though significant research has been conducted to look for an anti-angiogenesis agent from medicinal plants used in traditional Chinese medicine (TCM), most studies focus on VEGFR. Our findings demonstrate a potentially new therapeutic strategy of FA as an anti-tumor and anti-angiogenesis agent for melanoma by targeting a vital molecule in tumor angiogenesis. Through further research on the FA-mediated signal pathway after inhibition of FGFR1 activity, we showed that FA markedly inhibited the phosphorylation of PI3K and Akt in HUVEC. Furthermore, there are additional signaling proteins that are modulated by FGF1, including EKR and mTOR [26], playing key roles in tumor angiogenesis, but these pathways remains largely unaffected by FA treatment.

**Figure 9:**
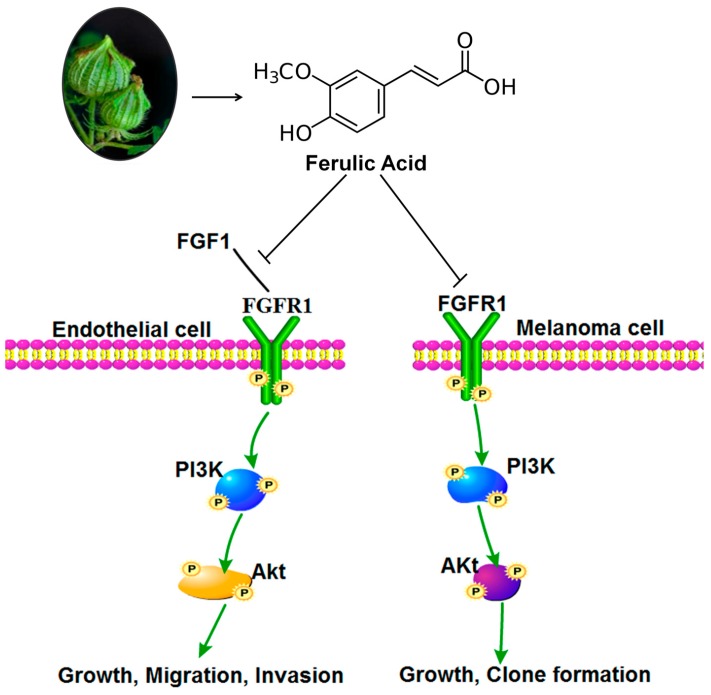
Proposed model by which ferulic acid treatment suppresses tumor angiogenesis and growth via inhibiting the FGFR1 signaling pathway.

Besides inhibiting tumor angiogenesis, FA also had a direct inhibitory effect on melanoma cell proliferation. FGF1 has been implicated in the pathogenesis of malignant melanoma. FA also attenuated the phosphorylation of PI3K and Akt, which are downstream regulatory proteins of FGFR1, indicating its ability to block the crucial oncogenic pathway. Clonogenic assays serve as a useful tool to test whether a given cancer therapy can reduce the clonogenic survival of tumor cells. Moreover, we showed that brief exposure to low-dose FA can reduce the growth and clonogenicity of melanoma cells *in vitro*. In addition, using a mouse xenograft model, we found that FA effectively suppresses tumor growth *in vivo*. Immunohistochemistry analysis showed that the expression of CD31, p-PI3K, p-Akt and p-FGFR1 proteins in xenografts was remarkably decreased, which suggested that FA inhibit tumor angiogenesis *in vivo*. Overall, our study indicated that FA at non-toxic dosages exerted potent anti-angiogenesis activities via specifically targeting FGFR1 and its signaling pathway in melanoma (Figure 9). FA combined with anti-angiogenic drugs will hopefully be a more effective treatment strategy for inhibiting tumor angiogenesis.

## 3. Experimental Section

### 3.1. Cell Culture and Reagents

Melanoma A375, CHL-1, SK-MEL-2, B16F10 cells and adult human epidermal melanocytes NHEM-a were purchased from Cell Resource Center, Shanghai Institutes for Biological Sciences. Cells were cultured in DMEM or 1640 with 10% fetal calf serum (FCS) (Gibco, Invitrogen, Carlsbad, CA, USA) and 1% penicillin/streptomycin mix (Gibco, Invitrogen, USA) and maintained at 37 °C in a humidified atmosphere containing 5% CO_2_. HUVEC were purchased from Chi Scientific (Maynard, MA, USA) and were cultivated in gelatinized culture plates in M199 medium supplemented with 15% FBS, 50 μg/mL endothelial cell growth supplement (ECGS, BD Biosciences, Qume Drive, San Jose, CA, USA) and 100 μg/mL heparin. Ferulic acid (98%, Sigma-Aldrich, St. Louis, MO, USA) was dissolved in dimethyl sulfoxide (DMSO; the final concentration is 0.1%) to prepare required concentrations [27]. FGF1 full-length protein was purchased from Abcam (Cambridge, MA, USA). The specific inhibitors for Akt (GSK690693) and PI3K (LY294002) were obtained from Selleck (Houston, TX, USA). The FGFR1 inhibitor SSR128129E (5 mg) was purchased from Selleck.

### 3.2. MTT Proliferation Assay

HUVEC (5 × 10^4^ cells/well) were plated onto a gelatinized 24-well culture plate and cultured in ECGS containing 15% FBS. HUVEC were treated with DMSO (0.1%) or different concentrations of FA (0, 2.5, 5, 10, 20, 30, 40 μM) for 24 h. Cell viability was determined by the MTT assay, as described previously [28]. After 4 h of incubation, the absorbance was measured at 450 nm with a microplate reader (Bio-Rad, Philadelphia, PA, USA). The results were calculated from six replicates of each experiment. Three independent experiments were performed.

### 3.3. Lactate Dehydrogenase Toxicity Assay

The LDH released into cell cultures is an index of cytotoxicity and the evaluation of the permeability of the cell membrane. HUVEC were seeded in a 96-well plate at a density of 5 × 10^4^ cells per well. After incubation with vehicle (0.1% DMSO), 1% Triton X-100 or various concentrations of FA for 24 h, cell supernatants were collected and analyzed for LDH activity using the LDH cytotoxicity assay kit from Keygen biotech. The absorbance of the formed formazan was read at 490 nm on a microplate reader [29].

### 3.4. Wound Healing

We examined the migration of HUVEC using a wound-healing assay. Briefly, cells were each grown on 3.5-cm plates with their respective culture media. After the growing cell layers had reached confluence, we inflicted a uniform wound in each plate using a pipette tip and washed the wounded layers with PBS to remove all cell debris. Then, we evaluated the closure at 24 h using bright-field microscopy [30].

### 3.5. Invasion Assay

The assay was performed with Matrigel-coated chambers from a BioCoat Matrigel Invasion Chamber Kit (BD Biosciences). Cells with 500 µL of serum-free medium were added into the upper chamber, and complete medium was added into the lower chamber. After incubation for 24 h, non-invasive cells in the upper surface of the membrane were removed, and the cells’ invasion to the lower surface of the membrane was fixed. Cell counting was then carried out by photographing the membrane through the microscope [25], and five random fields were taken.

### 3.6. Anchorage-Independent Growth Assay

Soft agar colony-formation assays were performed as previously described with minor modifications [19]. B16F10 (1 × 10^4^) cells in 1.5 mL of growth medium were mixed with 1.5 mL of 0.5% agarose in warmed growth medium containing vehicle (0.1% DMSO) or FA and layered on 0.5% base agar in 60-mm cell culture dishes. Culture medium containing scoparone was added only once; subsequently, medium without FA was added every week for 21 days until large colonies were evident. Cells were stained with crystal violet for colony counting.

### 3.7. Tube Formation Assay

The tube formation assay was performed using 12-well plates coated with 100 μL Matrigel basement membrane matrix (BD Biosciences) per well and polymerized at 37 °C for 30 min. HUVEC suspended in M199 medium containing 2% FBS were plated on the Matrigel at a density of 2 × 10^5^ cells/well. FA (2.5, 5 and 10 μM) was then added together with FGF1. After 6 h, The Matrigel-induced morphological changes were photographed, and the extent of capillary tube formation was evaluated by measuring the total tube length per field [31].

### 3.8. Rat Aortic Ring Assay

The rat aortic ring assay was performed as described previously [32]. In brief, 48-well plates were coated with 120 μL of Matrigel per well and polymerized in an incubator. Aortas isolated from 6-week-old male Sprague-Dawley rats were cleaned of periadventitial fat and connective tissues in cold phosphate-buffered saline and cut into rings of 1~1.5 mm in circumference. The aortic rings were randomized into wells and sealed with a 100-μL overlay of Matrigel. FGF1 in 500 μL of serum-free M199 with or without FA added into the wells, and the fresh medium was exchanged for every 2 day. After 6 day, microvessel sprouting was fixed and photographed using an inverted microscope (Olympus, Shanghai, China).

### 3.9. Chick Chorioallantoic Membrane Assay

The chick chorioallantoic membrane (CAM) assay was performed as described previously [33].

### 3.10. Western Blotting Assay

In brief, cell lysates were separated by 8% SDS-PAGE and transferred to polyvinylidene difluoride membranes. Membranes were then incubated with antibody against total-FGFR1 (1:1000, Abcam), phosphor-FGFR1 Y154 (1:1000, Abcam), phosphor-PI3K p85 Tyr458 (1:1000, Cell Signaling Technology, Danvers, MA, USA), total-PI3K p85 (1:1000, Cell Signaling Technology), phospho-Akt Thr308 (1:1000, Cell Signaling Technology), total-Akt (1:1000, Cell Signaling Technology), MMP-2 (1:1000, Abcam), MMP-9 (1:1000, Abcam), phosphor-FGFR2 Tyr463 (1:1000, Jiang Biological Technology Co., Ltd., Shanghai, China), total-FGFR2 (1:1000, Abcam), phosphor-mTOR Ser2448 (1:1000, Santa Cruz Biotech, Dallas, TX, USA.), total-mTOR (1:1000, Santa Cruz Biotech), phosphor-Erk1/2 Thr202/Tyr204 (1:1000, Santa Cruz Biotech), total-Erk1/2 (1:1000, Santa Cruz Biotech) and GAPDH (1:1000, Santa Cruz Biotech). After overnight incubation at 4 °C, membranes were incubated with horseradish peroxidase-conjugated IgGs (1:10,000, Bioworld Biotechnology, Louis Park, MN, USA). Immunoreactive bands were then visualized by the enhanced chemiluminescence (ECL) detection system (GE healthcare, Nanjing, China).

### 3.11. FGFR1 Kinase Inhibition Assay

The IC_50_ values for the inhibition of FGFR1 by FA were determined using a FRET-based *in vitro* kinase assay (Z’-lyte assay, Invitrogen, Paisley, UK). The kinase domains of FGFR1 was assayed in 50 mm HEPES pH 7.5, 0.01% C32H66O11 (BRIJ-35), 10 mm MgCl_2_, 2 mm MnCl_2_, 1 mm EGTA, 1 mm dl-Dithiothreitol (DTT), with 20 or 80 μm ATP, respectively. The assay was performed in triplicate in 384-well plates according to the manufacturer’s instructions [18].

### 3.12. FGFR Binding Assay

The FGFR binding assay was performed as described previously [34]. Briefly, FGF1 (50 ng/mL) in 50 μL of PBS was immobilized to 96-well plates. The wells were washed and blocked with 3% bovine serum albumin (BSA) in PBS for 2 h. FA with 1% BSA in PBS was added with FGFR1 (20 ng/mL; R&D Systems, Minneapolis, MN, USA) or FGFR2 (20 ng/mL; R&D Systems, Minneapolis, MN, USA) to FGF1-coated wells. After 3 h of incubation, the wells were washed thrice with PBST. FGFR1 or FGFR2 bound to FGF1 was determined by biotinylated anti-human IgG (Dako, Dako Denmark, Glostrup, Denmark) and horseradish peroxidase-conjugated streptavidin (Sigma), developed with tetramethylbenzidine substrate reagent (BD Biosciences) and quantified by measuring the absorbance at 450 nm.

### 3.13. Matrix Metalloproteins Activity Assay

The activity of MMP-9 and MMP-2 was determined by the QuickZyme MMPs activity assay (QucikZyme BioSciences, Cambridge, MA, USA) according to the manufacturer’s instructions. Briefly, after treatment, cells were washed with fresh medium and replaced with serum-free medium. After an additional 24 h, the medium was collected and centrifuged at 10,000× *g* for 10 min. The respective supernatant was added to the 96-well strip coated with MMP-9 antibody or MMP-2 antibody and incubated at 4 °C overnight. After washing with wash buffer 3 times, 50 µL assay buffer were added into the well, followed by adding 50 µL detection reagent. After incubation at 37 °C for 1 h, OD_405_ was measured with a Microplate Reader (Bio-Tek, Winooski, VT, USA).

### 3.14. Immunofluorescence Analysis

The effects of FA on FGF1-induced expression of FGFR1 phosphorylation in HUVEC were examined using an immunocytochemical method [35]. Cells were pretreated with or without FA for 24 h in the presence of FGF1. For immunofluorescent labeling, anti-p-FGFR1^Y154^ antibody was used as the primary antibody and goat anti-rabbit IgG-FITC (Santa Cruz Biotech) was used as a secondary antibody. Nuclei were counterstained with Hoechst 33258 (Biotime Biotech, Haimen, China). Fluorescent cells were observed and photographed under a laser scanning confocal microscope (LEICA TCS SP5, Mannheim, Germany).

### 3.15. Xenograft Models and Immunohistochemistry Detections

To build the melanoma xenograft, 3 × 10^6^ B16F10 cells were subcutaneously implanted into female C57BL/6 mice [36]. On the seventh day, mice appropriately-sized (150–300 mm^3^) tumors were divided randomly into six groups, including control group, positive drug group (Dacarbazine), vehicle-treated group and FA dosage groups. The mice were treated with FA or carboxy methylated cellulose (vehicle) daily by intragastric administration. Tumor volume and mice body weight were measured every 3 days. Tumor volume was calculated as mm^3^ = 0.5 × length (mm)^3^ width (mm)^2^. After euthanizing mice on Day 25, deparaffinized tumor sections were stained with specific antibodies, including CD31 (Abbiotec, Stockholm, Sweden), p-FGFR1^Y154^, p-PI3K p85^Tyr458^ and p-Akt^Thr308^. Detection was done with avidin-biotin-HRP complex (Thermo Scientific, Hudson, NH, USA) and diaminobenzidine as the chromogen [37]. Nuclei were counterstained with hematoxylin. All animal experiments were carried out in compliance with the Guidelines for the Zhejiang University School of Medicine (Animal Ethics Application Number SCX2013-0051. Approved by Animal Care and Use Committee of Zhejiang University School of Medicine at 15 January 2015).

### 3.16. Statistical Analysis

The data were presented as the mean ± SD. Differences in the results of two groups were evaluated using either two-tailed Student’s *t*-test or one-way ANOVA followed by *post hoc* Dunnett’s test. The differences with *p* < 0.05 were considered statistically significant.

## 4. Conclusions

In summary, our study indicated that FA exerted anti-angiogenesis activities at a non-toxic dosage via specifically targeting FGFR1 and its PI3K/Akt signaling pathway in melanoma. As a natural inhibitor against FGFR1, FA is a promising candidate for the development of anti-angiogenesis agents.

## Supplementary Materials

Supplementary materials can be found at http://www.mdpi.com/1422-0067/16/10/24011/s1.

## Abbreviations

HUVEC: human umbilical vein endothelial cell; FGF1: fibroblast growth factor 1; FGFR1: fibroblast growth factor receptor 1; VEGF: vascular endothelial growth factor; PIGF: placenta growth factor; PDGF: platelet-derived growth factor; PI3K: phosphatidyl inositol 3-kinase; Akt: protein kinase B; MMP: metal matrix proteinase; LDH: lactate dehydrogenase; CAM: chicken chorioallantoic membrane.
