# Supplementary material for: Ferulic Acid Exerts Anti-Angiogenic and Anti-Tumor Activity by Targeting Fibroblast Growth Factor Receptor 1-Mediated Angiogenesis
Source: Int J Mol Sci. 2015 Oct 12;16(10):24011–31. doi: 10.3390/ijms161024011 (PMC4632735; doi:10.3390/ijms161024011)
Supplement: Supplementary file 1 [file ijms-16-24011-s001.pdf]

# Supplementary Information

## 1. Materials and Methods

### *1.1. Immunoprecipitation Assay*

HUVEC were lysed in a culture dish by adding 0.5 mL of ice-cold RIPA lysis buffer. The supernatants were collected by centrifugation at 15,000× *g* for 10 min at 4 °C and then incubated with IgG (Cell Signaling Technology) or FGF1 in the presence or absence of FA at 4 °C overnight, followed by incubation with anti-FGF1 (Abcam) for 4 h. Then, supernatants were incubated with protein G-Sepharose (Santa Cruz) for 4 h. Following the removal of the supernatant by brief centrifugation (6000× *g*), the protein G-Sepharose was washed 3 times with lysis buffer and then boiled for 5 min in loading buffer. Immunoprecipitates were further analyzed by Western blotting using anti-FGFR1 antibody (1:1000, Abcam), anti-FGF1 antibody (1:1000, Abcam) and anti-FGFR2 (1:1000, Abcam).

### *1.2. Plasmids and Transfection*

The short small interfering RNA (siRNA) was constructed by Nanjing GenScript Biotechnology Co., Ltd. (Nanjing, China), with the sequence specifically targeted to the FGFR1 gene (5′-GAAGUGCAUACACCGAGAC-3′) and the FGFR2 gene (5′-GGACGGCACACCCUACGUU-3′). Transient transfection was performed using the Lipofectamine RNAi MAX reagent (Invitrogen) and following the manufacturer's instructions. After transfection, cell proliferation was measured by the MTT assay.

**Supplementary Figures**

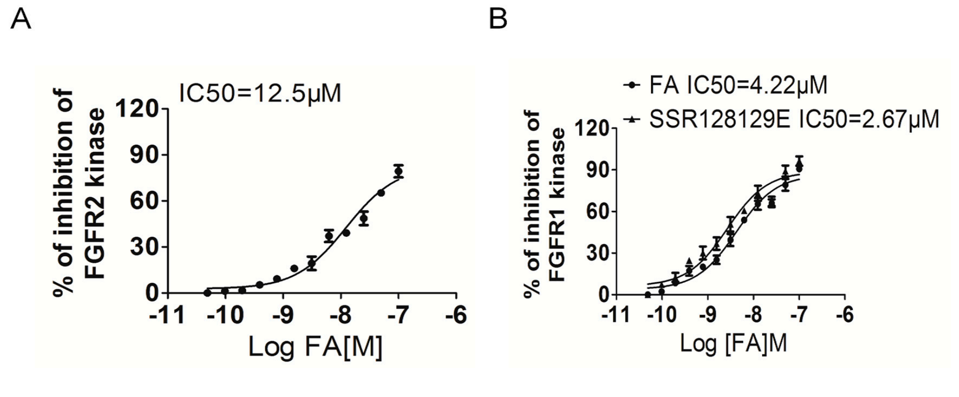


**Figure S1.** (**A**) Inhibition of FGFR2 kinase activity by FA was analyzed. Data are expressed as the percentages of the vehicle control. Data are from three independent experiments and are the mean ± SD. $n = 3$; (**B**) Inhibition of FGFR1 kinase activity by FA or SSR128129E was analyzed. Data are expressed as the percentages of the vehicle control. Data are from three independent experiments and are the mean ± SD. $n = 3$.

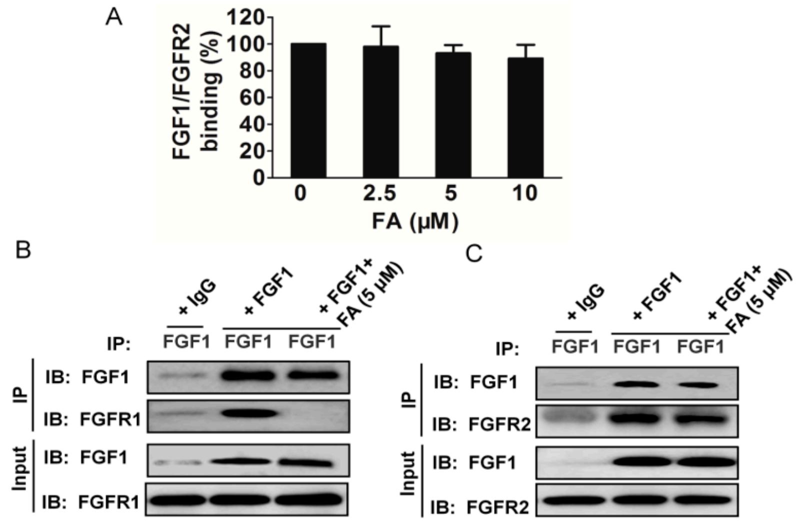


**Figure S2.** Effect of FA on the binding of FGFR2 to immobilized FGF1. (**A**) Data are from three independent experiments and are the mean ± SD. $n$ = 3; (**B**) FA inhibited FGF1-FGFR1 complex formation and did not interfere with FGF1 binding to FGFR2.

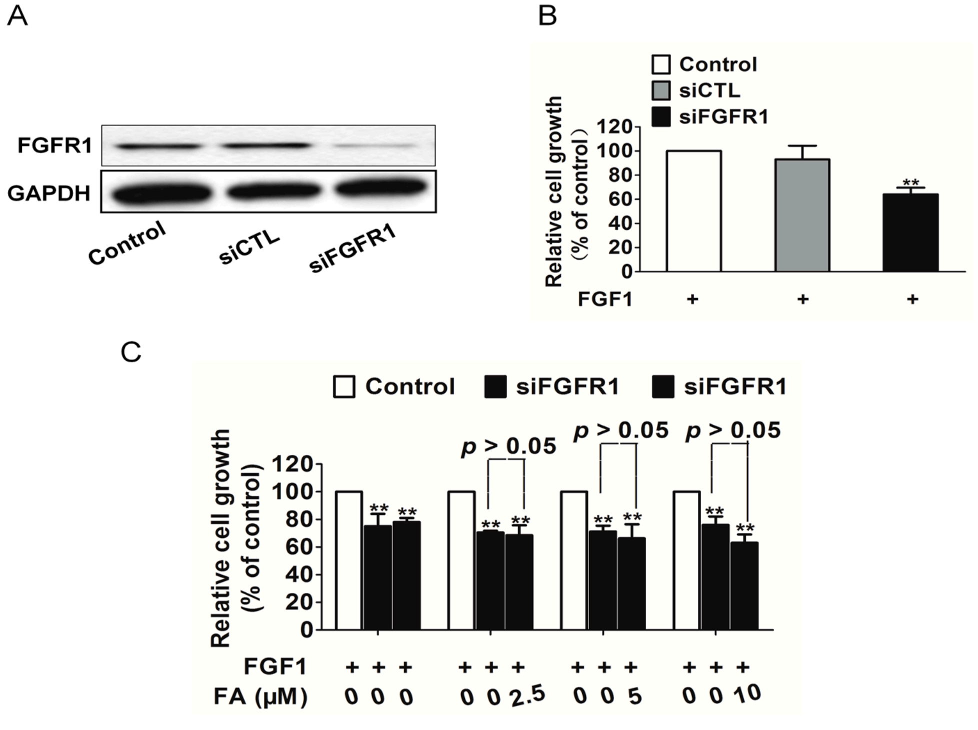


**Figure S3.** Effect of FA on the FGFR1 silencing HUVEC. (**A**) HUVEC were transfected with FGFR1 siRNA (0.15 μg/well for 96-well culture plates and 2 μg/well for six-well culture plates). Western blot shows that the FGFR1 was elevated in control cells, siCTL and siFGFR1-transfected cells. GAPDH was used as a loading control; (**B**) Proliferation assays indicated that FGFR1-siRNA and FGFR2-siRNA exerted inhibition on HUVEC proliferation *in vitro*. Data are from three independent experiments and are the mean ± SD. $n$ = 3, ** $p$ < 0.01 compared to the control; (**C**) The proliferation inhibitory effects of FA on HUVEC was abolished by FGFR1 siRNA. Data are from three independent experiments and are the mean ± SD. $n$ = 3, ** $p$ < 0.01 *vs.* the control.

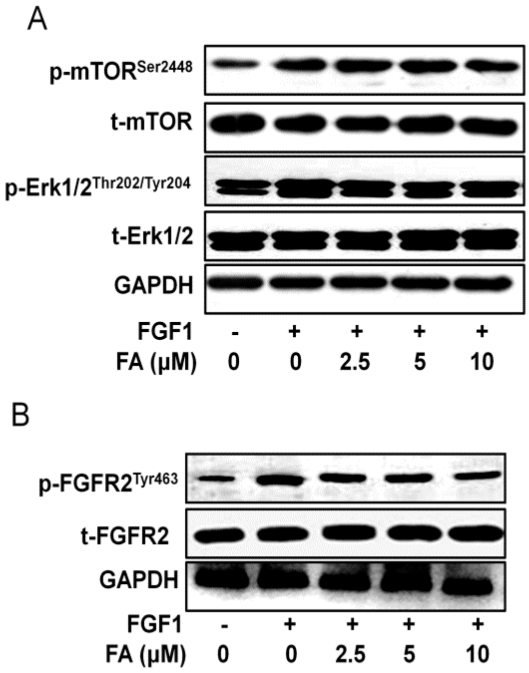


**Figure S4.** The effect of FA on FGF1-stimulated activity of ERK, mTOR and FGFR2 in HUVEC. (**A**) p-mTOR$^{Ser2448}$, t-mTOR, p-Erk1/2$^{Thr202/Tyr204}$ and t-Erk1/2 were measured in HUVEC after FA treated in the presence of FGF1. GAPDH expression was used as a loading control; (**B**) Total-FGFR2 and phosphorylation-FGFR2 were measured in HUVEC after FA treatment in the presence of FGF1. GAPDH expression was used as a loading control.

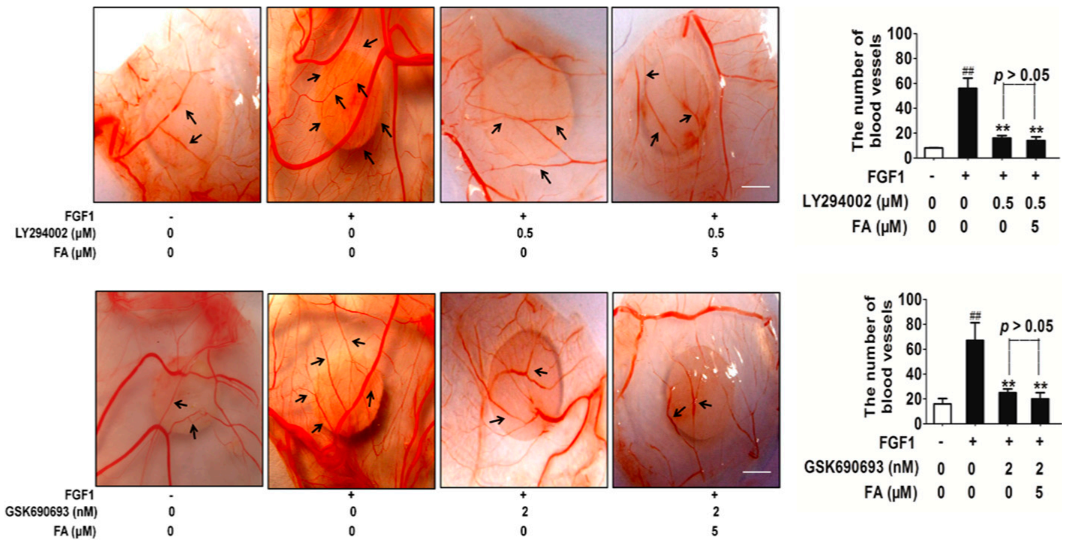


**Figure S5.** The effect of FA on angiogenesis dependent on FGFR1-mediated downstream signaling. In the presence of GSK690693 or LY294002, the CAM angiogenesis assay was conducted. Photo-pictographs of a typical experiment showing the angiogenesis pattern in different treatments. Arrows represent microvessel formation. The scale bar represents 0.5 cm. Data are from three independent experiments and are the mean ± SD. $n = 3$, $^{\#\#} p < 0.01$ compared to the control, ** $p < 0.01$ compared to the FGF1-alone treatment.

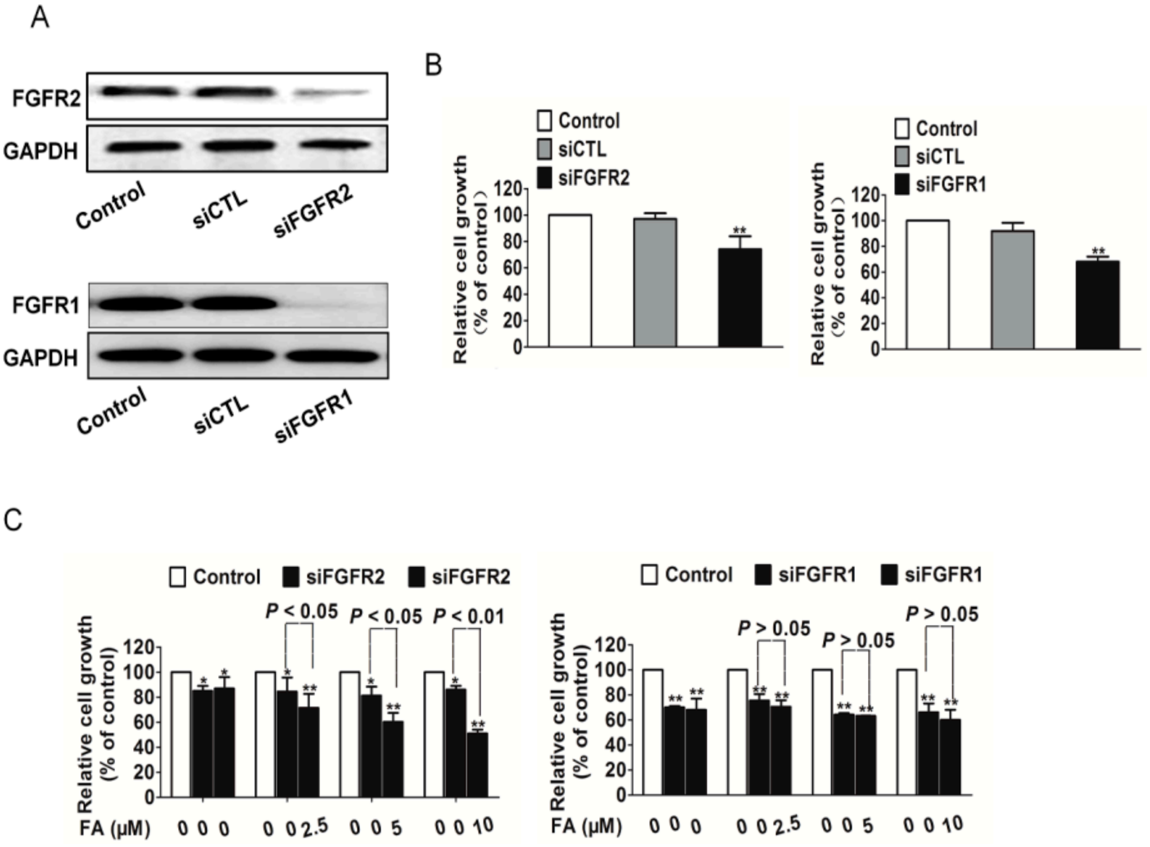


**Figure S6.** Effect of FA on the FGFR2 silencing or FGFR1 silencing B16F10 cells. (**A**) B16F10 cells were transfected with FGFR2 siRNA or FGFR1 siRNA (0.15 μg/well in 96-well culture plates for the MTT assay and 2 μg/well in six-well culture plates for Western blot assay). Western blot shows that the FGFR1 and FGFR2 were elevated in control cells, siCTL and siFGFR-transfected cells. GAPDH was used as the loading control; (**B**) Proliferation assays indicated that FGFR1-siRNA and FGFR2-siRNA exerted inhibition on B16F10 cell proliferation *in vitro*. Data are from three independent experiments and are the mean ± SD. $n = 3$, ** $p < 0.01$ compared to the control; (**C**) The proliferation inhibitory effects of FA on B16F10 were abolished by FGFR1 siRNA. Data are from three independent experiments and are the mean ± SD. $n = 3$, * $p < 0.05$; ** $p < 0.01$ *vs.* the control.
